# Comparative Efficacy of a High-Dose vs Standard-Dose Hepatitis B Revaccination Schedule Among Patients With HIV: A Randomized Clinical Trial

**DOI:** 10.1001/jamanetworkopen.2021.20929

**Published:** 2021-08-23

**Authors:** Jose Ignacio Vargas, Daniela Jensen, Felipe Martínez, Valeska Sarmiento, Felipe Peirano, Pedro Acuña, Felipe Provoste, Valentina Bustos, Francisca Cornejo, Antonieta Fuster, Martin Acuña, Felipe Fuster, Sabrina Soto, Denisse Estay, Werner Jensen, Rodrigo Ahumada, Juan Pablo Arab, Alejandro Soza, Francisco Fuster

**Affiliations:** 1 Hepatology Unit, Hospital Gustavo Fricke, Viña del Mar, Chile; 2 Gastroenterology Department, School of Medicine, Pontificia Universidad Católica de Chile, Santiago, Chile; 3 Internal Medicine and Endocrinology Department, Hospital Naval Almirante Nef, Viña del Mar, Chile; 4 Facultad de Medicina, Escuela de Medicina, Universidad Andrés Bello, Sede Viña del Mar, Viña del Mar; 5 Facultad de Medicina, Escuela de Medicina, Universidad de Valparaíso, Viña del Mar; 6 Laboratorio Clínico ACLIN, Viña del Mar, Chile; 7 Infectious Disease Unit, Hospital Gustavo Fricke, Viña del Mar, Chile

## Abstract

**Importance:**

Active immunization for hepatitis B virus (HBV) infection is recommended in patients living with HIV. Limited evidence is available about the most appropriate regimen of HBV vaccination among those who have not responded to an initial schedule.

**Objective:**

To determine the efficacy of a high-dose schedule compared with a standard dose of HBV vaccination.

**Design, Setting, and Participants:**

This double-masked, parallel-group, randomized controlled trial included patients living with HIV at a single outpatient HIV and hepatology clinic in Chile for whom previous HBV vaccination had failed. Patients with hepatitis B surface antibody (anti-HBs) titers less than 10 IU/L after an initial HBV vaccination regimen were included. Consecutive patients were recruited between December 2013 and March 2018. Data were analyzed in June 2018 using intention-to-treat analysis.

**Intervention:**

The high-dose HBV vaccination group consisted of 3 doses of 40 μg recombinant hepatitis B vaccine at 0, 1, and 2 months. The standard-dose group received 3 doses 20 μg each at 0, 1, and 2 months.

**Main Outcomes and Measures:**

Primary outcome was the serologic response to HBV vaccination (anti-HBs greater than 10 IU/L) 4 to 8 weeks after completion of the schedule. Secondary outcomes were anti-HBs greater than 100 IU/L and seroprotective anti-HBs at 1 year follow up.

**Results:**

A total of 107 patients underwent randomization (55 to the standard-dose group, 52 to the high-dose group); 81 (75.7%) were men, and the mean (SD) patient age was 47.0 (13.3) years. Nearly all patients were receiving antiretroviral therapy (105 patients [98%]) and 92 patients (86%) had an undetectable HIV viral load. Mean (SD) CD4 count was 418 (205) cells/mm^3^. There were no differences in baseline characteristics between groups. Serological response in the high-dose group was found in 36 of 50 patients (72%; 95% CI, 56.9%-82.9%) compared with 28 of 55 patients in the standard-dose group (51%; 95% CI, 37.1%-64.6%) (odds ratio, 2.48; 95% CI, 1.02-6.10; *P* = .03). Mean (SD) anti-HB levels were 398.0 (433.4) IU/L in the high-dose group and 158.5 (301.4) IU/L in the standard-dose group (*P* < .001). Of patients with a serological response in the high-dose group, 29 of 36 (80.6%) had anti-HBs titers greater than 100 IU/L compared with 14 of 28 responders (50.0%) in the standard-dose group (*P* = .02). At 1-year follow-up, 20 of 25 patients (80.0%) with a serological response in the high-dose group had protective anti-HBs vs 9 of 23 patients (39.1%) in the standard-dose group (*P* = .01).

**Conclusions and Relevance:**

The results of this randomized clinical trial suggest that use of a high-dose regimen for HBV revaccination for patients with HIV achieves a higher and longer-lasting serological response as compared with a standard-dose regimen.

**Trial Registration:**

ClinicalTrials.gov Identifier: NCT02003703

## Key Points

**Question:**

What is the efficacy of a high-dose vaccine schedule compared with standard dosage for hepatitis B virus (HBV) revaccination in patients living with HIV?

**Findings:**

In this randomized clinical trial including 107 adults at a single HIV and hepatology clinic in Chile, 72% of patients receiving a high dose of HBV vaccine achieved serological response as compared with 51% in the standard-dose group. Higher and longer-lasting hepatitis B surface antibody titers were seen in the high-dose group as well.

**Meaning:**

These results suggest that a high-dose regimen may be superior to a standard-dose schedule for HBV revaccination to achieve seroprotection in patients living with HIV.

## Teaser

This randomized clinical trial compares the standard hepatitis B virus (HBV) revaccination schedule for patients with HIV vs a regimen with higher doses.

## Introduction

People living with HIV are at risk of acquiring hepatitis B virus (HBV) infection because of common transmission mechanisms. HBV incidence is higher in patients with HIV than in the general population,^1^ and in patients with HIV the virus has a more aggressive course and higher rates of chronic infection, reactivation episodes, progression to cirrhosis, and hepatocellular carcinoma incidence.^2,3^ Therefore, HBV prevention is paramount,^4^ and screening and vaccination is recommended in all patients with HIV.^5,6,7^

The standard HBV vaccination schedule in immunocompetent patients considers 3 doses given at 0, 1, and 6 months.^8^ However, HBV vaccine response can be lower in people with HIV, ranging from 17% to 89% in previously published studies.^9^ Revaccination with a secondary regimen is recommended in patients not responding to a primary schedule.^6,7,10^ In patients with HIV, different revaccination schedules have been used to achieve seroprotection^9^ To date, there are limited data about the most appropriate schedule for revaccination, mostly from uncontrolled retrospective or cohort studies.^11^ Previous studies have reported response rates to HBV revaccination using diverse vaccination schedules considering standard dosage, shorter dose intervals,^12,13^ or double-dose at regular intervals^14,15^ (eTable 2 in Supplement 2). In an uncontrolled study, Cruciani et al^16^ used a high-dose regimen with a shorter interval between doses and reported high hepatitis B surface antibodies (anti-HBs) seroconversion rate. Clinical practice guidelines recommend the use of a high-dose schedule for HBV revaccination.^7,10^ However, a randomized clinical trial by Rey et al^17^ reported that the use of double-dose HBV vaccination was not superior to the standard-dose regimen in achieving seroprotection in patients with HIV that do not respond to an initial regimen. Complementarily, low rates of HBV immunization and completion of vaccination schedule have been described in people living with HIV who are suitable for immunization,^18^ and the use of a high-dose schedule of vaccination could be beneficial. Our study was aimed at evaluating the efficacy of a high-dose schedule of revaccination compared with a standard-dose regimen in patients with HIV who did not respond to an initial vaccination regimen in a randomized clinical trial.

## Methods

### Study Design and Participants

CORE-HIV (HBV Comparative Revaccination in HIV) is a phase 3 randomized clinical trial with parallel assignments and double masking that was conducted at Hospital Dr Gustavo Fricke, Viña del Mar, Chile, between December 2013 and March 2018. The institutional review board at the study center reviewed and approved the protocol, and written informed consent was obtained from each participant before enrollment. The study protocol (Supplement 1) was drafted following the Consolidated Standards of Reporting Trials (CONSORT) reporting guideline.

Adult patients (ie, aged over 18 years) living with HIV were eligible to participate irrespective of their viral load, CD4 counts, or antiretroviral therapy treatment strategies. To participate in this trial, a previous failed immunization against HBV using a standard dose of hepatitis B vaccine and negative serological markers for hepatitis B (hepatitis B surface antigens, hepatitis B core antigen antibodies [anti-HBc], and anti-HBs) were required. Failed initial immunization was defined as anti-HBs titers less than 10 IU/L checked 4 to 8 weeks after the HBV vaccination schedule was completed.^7,8^ In the initial vaccination schedule, anti-HBs serology and documentation of initial vaccination failure in most of the patients recruited for this trial was done in the context of a previous study by our group.^19^ No additional HBV vaccination doses were received by participants of this trial during the treatment or follow up period. Among exclusion criteria for the study were proven hypersensitivity to the vaccine or any of its components; a current diagnosis of a solid organ malignant neoplasm, decompensated chronic liver disease, chronic kidney disease, pregnancy, or unexplained fever in the last 7 days; and current treatment with systemic corticosteroids or other immunosuppressive medications.

### Data Collection

A basic clinical profile was obtained from every participant at baseline that included demographic and clinical features, clinical laboratory data, and characteristics regarding HIV infection. (Table 1; eTable 1 in Supplement 2). Blood samples were collected at baseline to assess for eligibility 4 to 8 weeks after the last dose of the vaccine was administered and at 1 year follow up for patients with a positive serological response.

**Table 1.** Patient Baseline Characteristics

Characteristic	No. (%)		
	Standard group (n = 55)	High-dose group (n = 52)	Total (n = 107)
Age, mean (SD), y	48.2 (12.9)	45.6 (13.7)	47.0 (13.3)
Sex			
Men	41 (74.5)	40 (76.9)	81 (75.7)
Women	14 (25.5)	12 (23.1)	26 (24.3)
BMI, mean (SD)	27.0 (5.1)	27.5 (4.2)	27.3 (4.7)
Alcohol consumption	31 (57.4)	27 (52.9)	58 (55.2)
Substance abuse	5 (9.3)	6 (11.5)	11 (10.4)
Active smoker	22 (40.7)	22 (43.1)	44 (41.9)
Diabetes	3 (5.6)	2 (4.0)	5 (4.8)
Arterial hypertension	16 (29.1)	12 (23.5)	28 (26.4)
Dyslipidemia	37 (69.8)	23 (46.9)	60 (58.8)
Syphilis	4 (7.3)	8 (16.0)	12 (11.4)
Hepatitis C virus infection	0	1 (2.0)	1 (0.9)
CD4 count, mean (SD), cells/mm^3^	424 (210)	412 (201)	418 (205)
Nadir CD4 count, (SD), cells/mm^3^	150.5 (17)	123.5 (17)	136.4 (115)
CD8 count, mean (SD), cells/mm^3^	887 (450)	1000 (497)	941 (474)
CD4/CD8 ratio, mean (SD)	0.58 (0.33)	0.49 (0.32)	0.54 (0.33)
Hepatitis C virus infection	0	1 (2.0)	1 (0.9)
Antiretroviral therapy	54 (98.2)	51 (98.0)	105 (98.1)
Undetectable viral load	49 (89.1)	43 (83.0)	92 (86.0)
Time living with HIV, median (IQR), mo	92 (46-158)	77 (46-122)	85 (46-142)
Time using ART, median (IQR), mo	58 (32-112)	55 (32-94)	56 (32-109)

Abbreviations: BMI, body mass index (calculated as weight in kilograms divided by height in meters squared); IQR, interquartile range.

### Randomization and Masking

Participants were randomized in a 1:1 ratio using permuted blocks of 10 patients in a computer algorithm to 1 of the 2 treatment arms. Randomization was not stratified and was carried out by an independent statistician who was also unaware of participant allocation. Allocation concealment was achieved by using sealed opaque envelopes. Candidate participants were screened for inclusion at their HIV outpatient clinic appointments.

### Interventions

Patients allocated to the high-dose dose arm received 3 doses of 40 µg each of recombinant hepatitis B vaccine (GlaxoSmithKline), which was administered immediately after randomization and 1 and 2 months afterwards. In this group, 1 dose of 20 µg was administered intramuscularly in 2 sites (ie, deltoid muscle) at each visit. Participants in the standard-dose arm received 3 doses of 20 µg each of the same vaccine using an identical dosing schedule. For this group, 1 dose of 20 µg was administered intramuscularly in 1 site at each visit.

### Primary and Secondary End Points

The primary end point for this randomized trial was a positive serologic response 4 to 8 weeks after completion of the intervention strategy. A positive response was defined as the presence of anti-HBs titers greater than 10 IU/L, as recommended by current guidelines.^8^ Secondary end points included the proportion of high-level responders, defined as patients who developed anti-HBs titers 100 IU/L or greater, the serological response at 1-year follow-up and the occurrence of local and systemic adverse events. Participants were required to stay in the HIV clinic for 2 hours after vaccination in order to detect immediate adverse reactions. A telephone interview was conducted to inquire for further adverse events 2 weeks after vaccination, and a survey was applied at a latter visit to further inquire for adverse reactions. Hospital admissions were monitored during the study period for up to 6 months to evaluate for possible systemic adverse reactions. Patients with a positive serological response for the primary outcome were followed for a repeat determination of anti-HBs titers 1 year after vaccination schedule completion to assess for long-term response.

### Laboratory Assays

The determination of hepatitis B surface antigens and quantification of anti-HBc and anti-HBs titers on serum samples were done using standardized assays (ie, anti-HBc reagent kit, electrochemiluminescence immunoassay anti-HBs reagent kit, electrochemiluminescence immunoassay, cobas e602 module) (Roche Diagnostics). Each sample was processed by technical laboratory staff masked to treatment group allocation.

### Statistical Analysis

Sample size calculations were initially made considering an estimated difference of 25% in the primary outcome (serologic response to HBV vaccination 4 weeks after completion of the schedule) between the 2 groups, to achieve a power of 80% at a 2-sided *P* < .05 significance level, indicating a sample size of 116 patients (58 per arm). An interim analysis with the results of the first 50 patients included in the study to assess for futility of the experimental treatment arm showed a nonstatistical difference of 26% in favor of the experimental arm, allowing us to adjust the sample size to 51 patients per group.

Descriptive statistics (eg, means, medians, proportions, interquartile ranges [IQRs]) were used to assess the characteristics of the study sample. The Fisher exact test was used to evaluate univariate association of categorical variables. Quantitative variables were compared using Mann-Whitney or *t* tests according to data distribution and variances. Ninety-five percent confidence intervals were constructed whenever appropriate. Binomial confidence intervals were estimated using the Clopper-Pearson method. All analyses were performed under the intention-to-treat principle. Missing data relevant to the primary and secondary outcomes were handled using multiple imputation techniques whenever appropriate (ie, when a proportion of missing data was greater than 5%^20^). Predictor variables were included in this procedure using linear regression for data showing normal distributions. Predictive mean matchings were preferred to impute data for variables with skewed distributions. It was planned to generate of 20 data sets in each case. All analyses were undertaken by a statistician who was unaware of participant allocation using Stata version 12.0 (StataCorp). The complete statistical analysis plan is detailed in Supplement 1.

## Results

**Figure 1.**
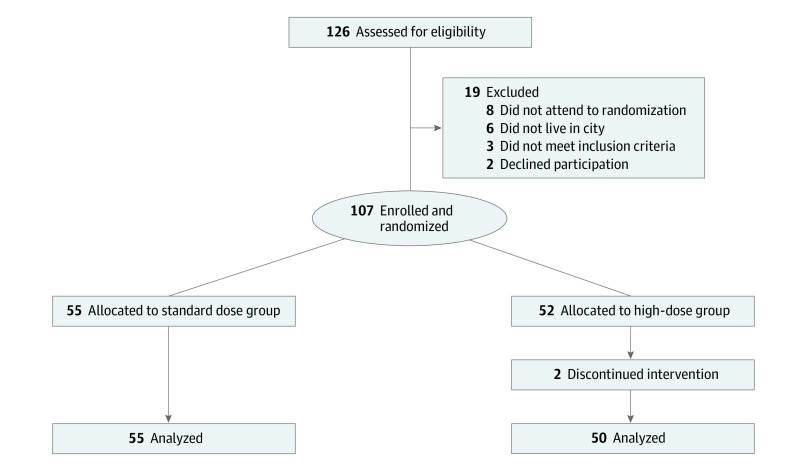
Study Flowchart

Patients were recruited between December 2013 and March 2018. Mean (SD) time between the completion of the initial HBV vaccination schedule and the randomization for this study was 16.8 (4.52) months (range, 7-23 months). A total of 126 patients were evaluated to enter the study and 107 were included and underwent randomization (55 assigned to the standard-dose arm, 52 assigned to the high-dose arm with the allocated intervention). No patients were lost to follow up in the standard-dose group and 2 patients discontinued the intervention and were lost to follow up in the high-dose arm (Figure 1).

Overall, patients included in the study had a mean (SD) age of 47 (13.3) years, and 81 of 107 patients (75.7%) were men. Only 1 patient had hepatitis C virus co-infection. Regarding HIV infection parameters, 105 patients (98%) were using antiretroviral therapy and 92 (86%) had an undetectable HIV viral load. Mean (SD) CD4 count was 418 (205). Median time for patients having the HIV diagnosis was 85 months (95% CI, 46-142 months) before entering the study. There were no differences in baseline patient characteristics between the intervention groups. Main patient characteristics are described in Table 1 and detailed baseline characteristics are depicted in (eTable 1 in Supplement 2).

**Figure 2.**
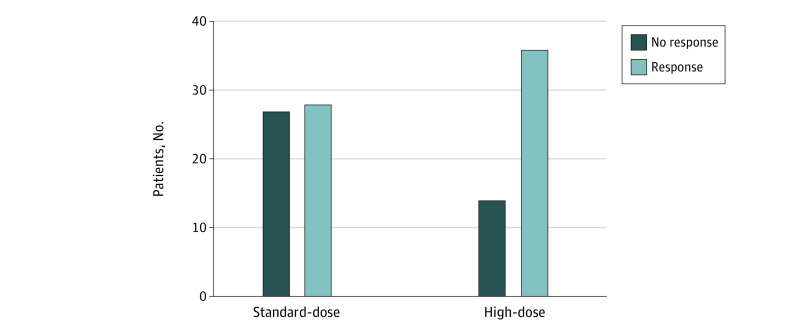
Comparison Between Serological Response Between Intervention Groups After Complete Vaccination Serological response was defined as hepatitis B surface antibody titers above 10 IU/L.

In intention-to-treat analysis, we found that the serological response in the standard-dose arm was 50.9% (28 of 55 patients) (95% CI, 41.4%-61.3%), which was significantly lower than the 72% (36 of 50 patients) (95% CI, 62.7%-80.6%) response in the high-dose group (OR, 2.48; 95% CI, 1.02-6.10; *P* = .03) (Figure 2).

**Figure 3.**
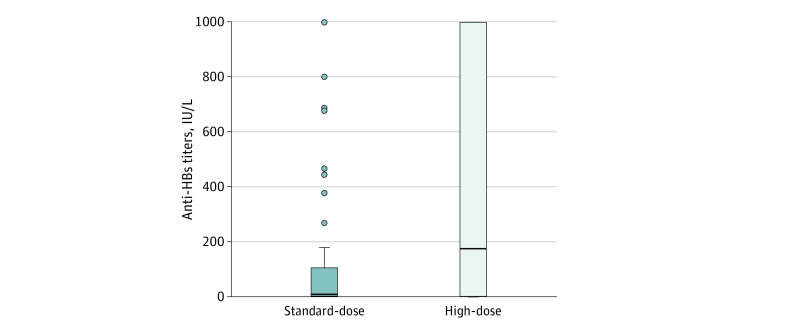
Hepatitis B Surface Antibody (Anti-HBs) Titers Comparison Between Intervention Groups Measured 4 to 8 Weeks After Vaccination Schedule Completion Bars indicate mean values; error bars, standard deviations; dots, numerical values.

The quantitative anti-HBs response was different between intervention groups as well. Mean (SD) anti-HBs titers at 4 to 8 weeks were 158.5 (301.4) IU/L in the standard-dose group and 398.0 (433.3) IU/L in the high-dose group (*P* < .001) (Figure 3). We also found significant differences in the proportion of patients with anti-HBs high-level response: 29 of 36 patients in the high-dose group had anti-HBs titers greater than 100 IU/L (80.6%; 95% CI, 77.5%-91.0%) compared with 14 of 28 patients in the standard-dose group (50.0%; 95% CI, 40.5%-60.4%; *P* = .02).

In patients who completed the 1-year follow-up, 20 of 25 patients (80%) with an initial positive serological response in the high-dose group had antibody titers in the protective range (ie, greater than 10 IU/L) compared with 9 of 23 patients (39%) in the standard-dose group (*P* = .01).

Local adverse reactions related to the injection site were seen only in 2 patients and no systemic reactions attributable to the vaccination were observed during the study period. Detailed results about the study outcomes considering intention-to-treat analysis are provided in Table 2.

**Table 2.** Study Outcomes

Characteristic	Group A (n = 55)	Group B (n = 50)	*P* value
Proportion of patients with vaccine response, No. (%)	28 (50.9)	36 (72.0)	.03^a^
Proportion of patients with high-level anti-HBs response, No. (%)	14 (50.0)	29 (80.6)	.02^a^
Hepatitis B antibody titer 4 wk, mean (SD)	158.5 (301.3)	397.6 (425.0)	.02^b^
Proportion of patients with vaccine response at 1 y, No. (%)	9 (39.1)	20 (80.0)	.007^a^
Hepatitis B antibody titer 1 y, mean (SD)	109.2 (283.4)	154.6 (273.0)	.57^b^

Abbreviation: anti-HBs, hepatitis B surface antibodies.

^a^ Determined using Fisher exact test.

^b^ Determined using *t* test.

## Discussion

The findings in our study suggest that the use of a high-dose schedule for HBV revaccination in patients living with HIV achieves a higher anti-HBs seroconversion rate and anti-HBs titers, as well as more frequent seroprotective anti-HBs at 1-year follow up, as compared with the use of a standard-dose regimen.

In our study, the seroconversion rate with anti-HBs seroprotective titers in the high-dose group (72%) using high-dose vaccination at 0, 1, and 2 months was similar to previous uncontrolled studies that have reported the response rate of double-dose vaccination schedules. Cruciani et al^16^ reported a 73% response to HBV revaccination in 26 patients with HIV prospectively followed using double-dose vaccination at 0, 1, and 2 months. Petit et al^15^ reported a 66.7% response to vaccination using double-dose at 0, 1, and 6 months in 30 patients in a retrospective study. Psevdos et al^14^ reported a response rate between 59% and 85% using a high-dose regimen at 0, 1, and 6 months in 101 retrospectively analyzed patients. Similarly, Rowley et al^21^ reported a 64% to 75% response to high-dose repeat HBV vaccinations. In their randomized clinical trial (the ANRS B-BOOST trial), Rey et al^17^ described a 74% response in a double-dose group using 0, 1, and 6 months interval dosage. Interestingly, the response with the use of a high-dose schedule with shorter intervals used in our study is also in accordance to the response described by Launay et al^22^ at week 12 after double-dose vaccination for primary HBV vaccination in their randomized study in patients with HIV. Our results are also similar to recent published studies using the same high-dose schedule for patients with cirrhosis, another subgroup of patients with lower response to HBV vaccination.^23^ Therefore, we could entail that the response rate of the high-dose schedule with shorter intervals between doses used in this study is comparable with previous studies using high doses for HBV revaccination in patients living with HIV.

On the other hand, the standard-dose group in our study had a 51% response to HBV revaccination. Looking at previously published data from studies using a standard-dose regimen, this response rate is higher than the 27% reported in Rowney et al^21^ and the 29% reported by Bloom et al,^24^ but lower than the 59% response reported by Psevdos et al^14^ and the 67% reported by Rey et al.^17^ The wide difference in the HBV vaccination response rate among studies using standard dosage could be related to several factors: study design, patient selection, intervals between vaccination doses, and type of vaccine used.^25^ Difference in patient selection, number or prior vaccine doses, and the use of a booster dose prior to randomization could explain the higher response in the ARNS B-BOOST trial^17^ as compared with the lower response with standard dosage found in our study. Given the lower rate of serological response of the standard HBV vaccination regimen at monthly intervals used in our study, this regimen should probably not be recommended for HBV revaccination in patients with HIV. Moreover, our findings support previously published evidence indicating that the serological response to a standard HBV schedule for revaccination in patients with HIV is possibly not optimal.

Definitions for the optimal seroprotective levels of anti-HBs in patients with HIV patients differs between guidelines, particularly when considering an anti-HBs titer of over 10 IU/L as minimal for seroprotection but a level greater than 100 IU/L as ideal.^6,7,10^ In our study, 80% of patients who responded to vaccination in the group with a high-dose schedule had a high-level response with anti-HBs greater than 100 IU/L as opposed to only 50% in the standard group. This percentage of patients with high-level anti-HBs response in the high-dose group is consistent with previous reports.^17^

Within our study, patients with a positive serological response were followed for 1 year after the vaccination schedule was completed, with important differences in the presence of seroprotective anti-HBs found between the intervention groups. Data about antibody titers and seroprotective levels in long-term follow up after HBV revaccination in patients with HIV are very limited. In a small sample, Rey et al^12^ reported 58.8% seroprotection after 1 year among patients with successful vaccination using variable vaccine doses. Cruciani et al^16^ described persistence of protective anti-HBs titers of 63% and 32.7% at 12 and 24 months of follow-up. Similar data are described in the RCT by Rey et al^17^ for follow up of anti-HBs titers at 72 weeks. As seen in our study, patients in the high-dose group had higher persistence of protective anti-HBs titers at 1-year follow up than was previously reported.

As anti-HBs titers wane over time, some guidelines suggest following patients with HIV annually with anti-HBs to assess for the need of booster HBV vaccine doses,^7,10^ although this recommendation is controversial and not included universally in surveillance guidelines.^6^ The findings in our study support the recommendation for antibody surveillance, especially if a patient has received standard-dose revaccination considering data herein showing that only 39% of these patients had seroprotective anti-HBs at 1-year follow up.

In previous studies, achieving HBV seroprotective titers has been associated with the completion of the vaccination schedule.^9^ However, in patients with HIV, a low rate of HBV vaccination^18^ and completion of schedule have been repeatedly reported.^26,27^ One of the factors that could influence this may be related to the usually long interval between doses for schedule completion.^28^ Strategies to increase the completion of the vaccination schedule are urgently needed. The use of a vaccination schedule with a shorter interval between doses, as the one used in our study, could be appropriate to that purpose.

### Limitations

Our study has several limitations. First, this is a single-center study in an outpatient clinic that included predominantly male sex patients because of the intrinsic characteristics of the HIV clinic at our institution, which may affect the external validity of the study results. Second, the standard-dose arm in our study considered simple doses at monthly intervals, which can differ from institutions where simple doses at 0, 1, and 6 months are in practice. Third, there was a moderately low response in the standard-dose treatment arm, still in accordance with previously published studies, but this could factor into the statistical significance of the results between groups. However, sample size calculations were estimated to assess for a difference of this magnitude.

## Conclusions

To the best of our knowledge, this is the first randomized clinical trial to use a high-dose schedule with a shorter interval between doses compared with a standard-dose regimen for HBV revaccination in patients with HIV who were nonresponders to an initial HBV vaccination schedule, demonstrating that the use of a high-dose schedule for revaccination achieved a higher prevalence of patients with seroprotective anti-HBs levels, a more robust serological response, and a longer-lasting antibody response as evaluated at 1-year follow up.

 As such, we believe that the high dose with shorter interval schedule used in this study could be considered as one of the primary options in stable patients with HIV for HBV revaccination. In line with our findings, we suggest that a standard-dose regimen at monthly intervals should probably not be recommended for these patients. The persistent serological protection 1 year after vaccination in the high-dose schedule group further supports the use of this high-dose HBV revaccination schedule in patients with HIV.

## Notes

Supplement 1.
Trial Protocol and Statistical Analysis Plan

Supplement 2. **eTable 1.** Detailed Patient Baseline Characteristics **eTable 2.** Summary of Previously Published Studies for HBV Re-Vaccination in Patients Living With HIV

Supplement 3.
Data Sharing Statement
