# Supplementary material for: Comparative Efficacy of a High-Dose vs Standard-Dose Hepatitis B Revaccination Schedule Among Patients With HIV: A Randomized Clinical Trial
Source: JAMA Netw Open. 2021 Aug 23;4(8):e2120929. doi: 10.1001/jamanetworkopen.2021.20929 (PMC8383137; doi:10.1001/jamanetworkopen.2021.20929)
Supplement: Supplement 1. — Trial Protocol and Statistical Analysis Plan [file jamanetwopen-e2120929-s001.pdf]

# Comparative efficacy of an intensified re-vaccination scheme for Hepatitis B virus infection among patients infected with HIV (CORE-HIV): A Randomized Controlled Trial. Study protocol

## Investigator Name & Contact Information

Francisco Fuster MD.
Hospital Gustavo Fricke, Clínica Reñaca. Anabaena 363. Viña del Mar. Chile.
Business phone: 56322891219
Business mail: fusterf@vtr.net
Jose Ignacio Vargas, MD
Hospital Gustavo Fricke, Clínica Reñaca. Anabaena 363. Viña del Mar. Chile.
Business phone: 56322891219
Business mail: jivargasd@uc.cl

## Institution Name

Hospital Gustavo Fricke, Viña del Mar, Chile.

## Study Title:

Comparative Efficacy of an Intensified Re-vaccination Scheme for Hepatitis B Virus Infection Among Patients Infected With HIV: A Randomised Trial (CORE-HIV)

## Study Rationale:

Vaccination against hepatitis B is commonly prescribed among patients living with HIV. However, current research has shown a lower response to this vaccine, which often ranges between 34% to 65%. Several guidelines have recommended revaccination for non-responders, but many uncertainties remain regarding specific schedules and dosing schemes. This situation is partially explained in the lack of randomised trials comparing different vaccination strategies. The purpose of this study is to determine the effectiveness of an intensified immunisation schedule as contrasted to a standard-dose one in increasing the serological response to the hepatitis B virus. Revaccination is recommended at monthly intervals.

Arm 1: standard-dose vaccine at 0, 4 and 8 weeks.

Arm 2: double-dose vaccine at 0, 4 and 8 weeks.

## Hypothesis

¿Among patients living with HIV and with a failed serological response to a standard hepatitis-B virus vaccine, does the use of an intensified strategy (40mcg) as compared to a standard revaccination (20mcg) increase serological response rates?

H0: Nonresponding patients living with HIV will not have a different response rate to an intensified scheme of the hepatitis B virus vaccine as compared to a standard strategy.

| |
|---|
| H1: Nonresponding patients living with HIV will have a different response rate to an intensified scheme of the hepatitis B virus vaccine as compared to a standard strategy. |
| Primary Objective(s) / Endpoint(s) |
| Serologic Response, defined as presence of anti-HBs greater than 10 ui/ml, 4-8 weeks after completion of the allocated vaccination scheme. |
| Secondary Objective(s) / Endpoint(s) |
| - Serological response of anti-HBs >100 UI/L.<br>- Serological response at 1 year follow up<br>- Determine the frequency of local adverse reactions to the vaccine up to one week after exposure.<br>- Development of any systemic adverse reaction attributable to vaccination. |
| Treatment |
| Arms 1: experimental. Patients allocated to this arm will receive three doses of 40 mcg each of recombinant hepatitis B vaccine (Engerix-B ®). Doses will be administered at 0, 1 and 2 months.<br>Arm 2: Active comparator. Patients allocated to this arm will receive three doses of 20 mcg each of recombinant hepatitis B vaccine (Engerix-B ®). Doses will be administered at 0, 1 and 2 months. |
| Trial Population |
| Patients with HIV who fail to respond to a primary hepatitis B series vaccine from the outpatient clinic of the Infectology Unit of Hospital Gustavo Fricke, Viña del Mar, Chile. Older than 18 years of age, both genders. |
| Sample Size and Sample Size Justification |
| Sample size calculations were made considering an estimated difference of 25% in the primary outcome (serologic response to HBV vaccination 4 weeks after completion of the schedule) between the two groups, to achieve a power of 80% at a two-sided 0.05 significance level. As such, a sample size of 116 patients (58 per arm) was calculated. |
| Key Inclusion Criteria |
| Older than 18 years of age.<br>Patients infected with Human Immunodeficiency Virus (HIV)<br>Failed previous vaccination with a standard dose scheme of recombinant hepatitis B vaccine (20mcg at 0, 1 and 6 months). Nonresponders will be considered as those |

patients presenting a hepatitis B surface antigen antibody titer lower than 10UI/mL 4 to 8 weeks after the last dose of the vaccine.
Provision of informed consent.

## Key Exclusion Criteria

Proven Hepatitis B virus infection (acute or chronic).
Proven hypersensitivity to the vaccine or any of its components.
Current diagnosis of a solid organ neoplasia, decompensated chronic liver disease, chronic kidney disease, pregnancy or unexplained fever in the last 7 days were excluded from the trial. Patients undergoing treatment with systemic corticosteroids or other immunosuppresive medications were excluded as well.

## Study Assessments

- Primary Outcome Measure: Serologic Response, 4 to 8 weeks after completion of the vaccination schemes.
- Secondary Outcome:
- Anti-HBs titres >100 UI/L.
- Serological response at 1 year follow up
- Safety Measures: local and systemic adverse reactions after each dose of vaccine.

## Data and Statistical Plan

First, descriptive statistics will be performed to assess the characteristics of the study sample. The Fisher's exact test will be used to evaluate the bivariate association of categorical variables. Quantitative variables will be compared using Mann–Whitney's or Student's t-tests according to data distribution characteristics and variances, which will be assessed using histograms.

Analyses aimed to determine factors associated with a positive serological response will be performed using multivariate logistic regression techniques. Lineality of included variables will be assessed graphycally, and every included model will evaluate the existence of potential interactions during development. The final model's diagnostic accuracy will be established using ROC curves. Goodness of fit will be assessed using Hosmer's an Lemeshow's test.

This analysis plan will be performed by a statistician who will be unaware of both treatment allocation and clinical assessment of included patients using Stata v10.0® (StataCorp, 1996–2011).

## References

1. Kaplan JE, Benson C, Holmes KH, et al. Guidelines for prevention and treatment of opportunistic infections in HIV-infected adults and adolescents: recommendations from CDC, the National Institutes of Health, and the HIV Medicine Association of the Infectious Diseases Society of America. MMWR Recomm Rep 2009; 58:1
2. Rockstroh JK, Bhagani S, Benhamou Y, et al. European AIDS Clinical Society (EACS) guidelines for the clinical management and treatment of chronic hepatitis B and C coinfection in HIV-infected adults. HIV Med 2008; 9:82

3. Geretti AM, Brook G, Cameron C, et al. British HIV Association guidelines for immunization of HIV-infected adults 2008. HIV Med 2008; 9: 795–848.
4. Jennifer A Whitaker, Nadine G Rouphael, Srilatha Edupuganti, Lilin Lai, Mark J Mulligan. Strategies to increase responsiveness to hepatitis B vaccination in adults with HIV-1. Lancet Infect Dis 2012; 12: 966–76
5. Rey D, Krantz V, Partisani M, et al. Increasing the number of hepatitis B vaccine injections augments anti-HBs response rate in HIV-infected patients. Effects on HIV-1 viral load. Vaccine 2000; 18: 1161–65.
6. Pettit NN, DePestel DD, Malani PN, Riddell J 4th. Factors associated with seroconversion after standard dose hepatitis B vaccination and high-dose revaccination among HIV-infected patients. HIV Clin Trials 2010; 11: 332–39.
7. Cruciani M, Mengoli C, Serpelloni G, et al. Serologic response to hepatitis B vaccine with high dose and increasing number of injections in HIV infected adult patients. Vaccine 2009; 27: 17–22.
8. de Vries-Sluijs TE, Hansen BE, van Doornum GJ, et al. A prospective open study of the efficacy of high-dose recombinant hepatitis B rechallenge vaccination in HIV-infected patients. J Infect Dis 2008; 197: 292–94.
9. Bloom A, Jackson K, Kiviat A, Zheng H, Sax P, Gandhi R. Repeat hepatitis B vaccination may lead to seroprotection in HIV-infected patients who do not respond to an initial series. J Acquir Immune Defic Syndr 2009; 50: 110–13.
10. Psevdos G, Kim JH, Groce V, Sharp V. Efficacy of double-dose hepatitis B rescue vaccination in HIV-infected patients. AIDS Patient Care STDS 2010; 24: 403–07.
