# Supplementary material for: Comparative Efficacy of a High-Dose vs Standard-Dose Hepatitis B Revaccination Schedule Among Patients With HIV: A Randomized Clinical Trial
Source: JAMA Netw Open. 2021 Aug 23;4(8):e2120929. doi: 10.1001/jamanetworkopen.2021.20929 (PMC8383137; doi:10.1001/jamanetworkopen.2021.20929)
Supplement: Supplement 2. — eTable 1. Detailed Patient Baseline Characteristics eTable 2. Summary of Previously Published Studies for HBV Re-Vaccination in Patients Living With HIV [file jamanetwopen-e2120929-s002.pdf]

Supplemental Online Content

Vargas JI, Jensen D, Martínez F, et al. Comparative efficacy of a high-dose vs standard dose hepatitis B revaccination schedule among patients with HIV. *JAMA Netw Open.* 2021;4(8):e2120929. doi:10.1001/jamanetworkopen.2021.20929

eTable 1. Detailed Patient Baseline Characteristics

eTable 2. Summary of previously published studies for HBV re-vaccination in patients living with HIV

This supplemental material has been provided by the authors to give readers additional information about their work.

© 2021 Vargas JI et al. *JAMA Network Open.*

eTable 1. Detailed Patient Baseline Characteristics.

Table 2: Detailed Patient Baseline Characteristics				
Characteristic	Standard Group (n=55)	Intensified Group (n=52)	Total (n=107)	P-Value
Clinical Characteristics				
Mean age (years) (SD)	48.2±12.9	45.6±13.7	47.0±13.3	0.31 [1]
Male sex (n, %)	41 (75.9%)	40 (78.4%)	81 (75.7%)	0.82 [2]
Mean weight (Kg) (SD)	75.2±21.8	76.1±14.8	75.7±18.5	0.81 [1]
Mean body mass index (Kg/m2) (SD)	27.0±5.1	27.5±4.2	27.3±4.7	0.68 [1]
Mean abdominal perimeter (cm) (SD)	94.3±10.9	95.4±13.9	94.8±12.2	0.78 [1]
Alcohol consumption (n, %)	31 (57.4%)	27 (52.9%)	58 (55.2%)	0.70 [2]
Substance abuse (n, %)	5 (9.3%)	6 (11.5%)	11 (10.4%)	0.76 [2]
Active smoker (n, %)	22 (40.7%)	22 (43.1%)	44 (41.9%)	0.85 [2]
Diabetes mellitus (n, %)	3 (5.6%)	2 (4.0%)	5 (4.8%)	1.00 [2]
Arterial hypertension (n, %)	16 (29.1%)	12 (23.5%)	28 (26.4%)	0.67 [2]
Dyslipidemia (n, %)	37 (69.8%)	23 (46.9%)	60 (58.8%)	0.03 [2]
Syphilis (n, %)	4 (7.3%)	8 (16.0%)	12 (11.4%)	0.22 [2]
Hepatitis C virus infection (n, %)	0 (0%)	1 (2.0%)	1 (1%)	0.47 [2]
Laboratory				
Mean serum creatinine (mg/dl) (SD)	0.81±0.13	0.80±0.14	0.81±0.14	0.86 [2]
Mean blood glucose (mg/dl) (SD)	96.8±27.4	95.5±34.4	96.2±30.9	0.82 [2]
Mean thyrotropin (uUI/mL) (SD)	3.5±1.9	3.1±2.0	3.3±1.9	0.34 [1]
Mean cholesterol (mg/dl) (SD)	206.7±45.0	197±37.8	201±41.6	0.23 [1]
Mean LDL cholesterol (mg/dl) (SD)	113±44.2	127±50.1	120.6±47.6	0.15 [1]
Mean HDL cholesterol (mg/dl) (SD)	47.2±15.5	45.9±14.1	46.6±14.7	0.67 [1]
Mean triglycerides (mg/dl) (SD)	227.5±138	202.3±167	215±152	0.40 [1]
Mean bilirubin (mg/dl) (SD)	0.8±0.7	1.0±1.1	0.90±0.91	0.28 [1]
Mean glutamic pyruvic transaminase (U/L) (SD)	34.4±19.9	37.6±20.5	36.0±20.1	0.41 [1]
Mean glutamic oxoalacetic transaminase (U/L) (SD)	29.4±11.5	30.6±9.8	30.0±10.6	0.57 [1]
Mean gammaglutamyl transpeptidase (U/L) (SD)	52.3±52.5	50.2±43.1	51.4±48.1	0.84 [1]
Mean alkaline phosphatase (U/L) (SD)	110±33.1	108.2±34.4	109.1±33.6	0.78 [1]
Mean prothrombin time (%) (SD)	98.7±13.4	98.2±15.7	98.5±14.5	0.88 [1]

© 2021 Vargas JI et al. *JAMA Network Open.*

Mean serum albumin (g/dL) (SD)	4.4±0.48	4.4±0.39	4.4±0.42	0.93 [1]
Mean hematocrit (%) (SD)	43.3±4.5	43.5±3.4	43.4±4.0	0.74 [1]
Mean leucocytes (x1000cells/mm3) (SD)	6.5±2.5	6.6±2.0	6.6±2.3	0.67 [1]
Mean platelets (x1000cells/mm3) (SD)	250±63	238±55	244±60	0.29 [1]
Mean CD4 count (cells/mm3) (SD)	424±210	412±201	418±205	0.78 [1]
Nadir CD4 count (cells/mm3) (SD)	150.5±17	123.5±17	136.4±115	0.23 [3]
Mean CD8 count (cells/mm3) (SD)	887±450	1000±497	941±474	0.22 [1]
Mean CD4/CD8 ratio (SD)	0.58±0.33	0.49±0.32	0.54±0.33	0.17 [1]
HIV Infection Characteristics				
Hepatitis C Virus infection (n, %)	0 (0%)	1 (2.0%)	1 (1%)	0.47 [2]
HIV Stage (n, %)				0.92 [2]
A1	2 (3.9%)	2 (3.9%)	4 (3.9%)	
A2	7 (13.73%)	13 (25.5%)	20 (19.6%)	
A3	4 (7.8%)	4 (7.8%)	8 (7.8%)	
B1	1 (2%)	0 (0%)	1 (1%)	
B2	5 (9.8%)	6 (11.8%)	11 (10.8%)	
B3	13 (25.9%)	10 (19.6%)	23 (22.6%)	
C1	1 (2%)	1 (2%)	2 (2%)	
C2	1 (2%)	1 (2%)	2 (2%)	
C3	17 (33.3%)	14 (27.5%)	31 (30.4%)	
Antiretroviral Therapy (n, %)	54 (98.2%)	51 (98.0%)	105 (98.1%)	1.00 [2]
Use of Efavirenz (n, %)	25 (47.2%)	26 (51.0%)	51 (49.0%)	0.85 [2]
Undetectable viral load (n, %)	49 (89.1%)	43 (83.0%)	92 (86.0%)	0.41 [2]
Median time living with HIV (months) (IQR)	92 (46-158)	77 (46-122)	85 (46-142)	0.50 [3]
Median time using ART (months) (IQR)	58 (32-112)	55 (32-94)	56 (32-109)	0.89 [3]

© 2021 Vargas JI et al. *JAMA Network Open.*

eTable 2. Summary of previously published studies for HBV re-vaccination in patients living with HIV.

Study	Year	Country	Design	N	Vaccination schedule (months)	Response (%)	Ref.
Rey et al.	2000	France	Prospective	9	Double<br>0-1-2	78	(13)
de Vries- Sluijs et al.	2008	Netherlands	Prospective	144	Double<br>0-1-2	50.7	(14)
Cruciani et al.	2009	Italy	Prospective	26	Double<br>0-1-2	73	(17)
Bloom et al.	2009	USA	Retrospective	63	Variable	29-36	(25)
Pettit et al.	2010	USA	Retrospective	30	Double<br>0-1-6	66.7	(16)
Psevdos et al.	2010	USA	Retrospective	101	Simple vs Double<br>Variable	Simple: 59<br>Double: 85	(15)
Rowley et al.	2014	UK	Prospective	38	Variable	27-75	(22)
Rey et al.	2015	France	RCT	178	Simple vs Double<br>0-1-6	Simple: 67%<br>Double: 74%<br>NS (*).	(18)

© 2021 Vargas JI et al. *JAMA Network Open.*
