# Supplementary material for: Comparative Efficacy of a High-Dose vs Standard-Dose Hepatitis B Revaccination Schedule Among Patients With HIV: A Randomized Clinical Trial
Source: JAMA Netw Open. 2021 Aug 23;4(8):e2120929. doi: 10.1001/jamanetworkopen.2021.20929 (PMC8383137; doi:10.1001/jamanetworkopen.2021.20929)
Supplement: Supplement 3. — Data Sharing Statement [file jamanetwopen-e2120929-s003.pdf]

Vargas JI, Jensen D, Martínez F, et al. Comparative efficacy of a high-dose vs standard dose hepatitis B revaccination schedule among patients with HIV. *JAMA Netw Open*. 2021;4(8):e2120929. doi:10.1001/jamanetworkopen.2021.20929

Data Sharing Statement

Data

Data available: Yes
Data types: Deidentified participant data
How to access data: jivargasd@uc.cl
When available: With publication

Supporting Documents

Document types: None

Additional Information

Who can access the data: researchers whose proposed use of the data has been approved
Types of analyses: Specified purpose
Mechanisms of data availability: after approval of a proposal
Any additional restrictions: no.
